# Brain and self – a neurophilosophical account

**DOI:** 10.1186/1753-2000-7-28

**Published:** 2013-07-31

**Authors:** Georg Northoff

**Affiliations:** 1 Director of the Mind, Brain Imaging and Neuroethics Research Unit, Institute of Mental Health Research, Royal Ottawa Mental Health Centre, 1145 Carling Avenue, Ottawa, Canada

## Abstract

We have experience and are conscious of the world. Who though is conscious? This is the subject or self of experience. While in the past the concept of self has been matter of philosophical discussion, psychoanalysis shifted it into the domain of psychology where it surfaced as ego. More recently, brain imaging allows to investigate the neural mechanisms underlying our subjective experience of a self. The article focuses on discussing different concepts of self as based on the philosophical accounts. These are then complemented by neuroscientific data on self and self-reference. Finally both philosophical and neuroscientific accounts are directly compared with each other while at the same time their relevance for psychoanalysis of self and ego are pointed out.

## Introduction: concept of self

You read these lines. You find them boring and your experience is thus signified by boredom. Who experiences this boredom? You. You are the subject of the experience of boredom. Without you as subject of this experience, you could not experience anything at all, not even boredom. This subject of experience has been described as the ‘self’. It is your ‘self’ that makes it possible for you to experience things. The self is a necessary condition for the possible constitution of experience and thus also consciousness. It is clear therefore that there is much at stake when it comes to the self. We thus need to discuss how to characterize and define the concept of self. Why is the self so important? Because we usually assume that somebody must have consciousness. Somebody speaks a language. And somebody acquires a second language when coming for instance to a new country. Without somebody we may remain unable to do all these things. Who though is this somebody? This is what is traditionally called self. Hence the self is of central relevance. Who though is this self? This is the topic of the present contribution. In what follows, I will outline 4 ways of conceptualising the self; the mental self, the empirical self, the phenomenal self and the minimal self. I will then consider how the self can and has been researched experiementally in respect to the brain before concluding with a discussion on the concept of self and its relation to identity and the environment.

## Concept of self

This section introduces four different concepts of self. The mental self is supposed to be based on our thoughts and a specific mental substance. This is different in the concept of the empirical self. Here the self is assumed to be no longer based on a mental substance but rather on representing and reflecting about the biological processes in the own body and brain. Another concept of self, the phenomenal self, starts from what we can experience in our consciousness. In addition to the content like this book in front of me, consciousness comes with an awareness of the own self, pre-reflective self-awareness or phenomenal self. Finally, most recently philosophers speak of a minimal self that is supposed to be based on our body and its physiological processes.

### Concept of self: mental self

What is the self? What must it look like in order to presuppose experience and be the subject of our experience? The nature of the self has often been determined as a specific ‘thing’. Stones are things, the table on which your laptop stands is a thing. And in the same way the table makes it possible for the laptop to stand on, the self may be a thing that makes experience and consciousness possible. In other words, these, metaphorically speaking, stand on the shoulders of the self. However, another question is whether the self is a thing or, as philosophers such as Rene Descartes suggest, a substance? A substance is a specific stuff, entity or material that is supposed to serve as basis for something like a self. For instance the body can be traced back to a physical substance while the self is associated with mental stuff, e.g., mental substance.

Is our self real and thus exists? Or is it just an illusion? Let’s compare the situation to perception. When we perceive something in our environment, we sometimes perceive not a real thing but an illusion which in reality does not exist. What though exists and is real? This is what philosophers call a metaphysical question, the one for existence and reality. Earlier philosophers like Rene Descartes assumed that the self is real and exists. However, he also assumed that the self is different from the body. Hence self and body exists but differ in their existence and reality. The self can thus be not a physical substance but rather a mental substance: It is a feature not of the body but of the mind and thus a mental entity rather than physical substance.

However, the characterization of the self as mental entity has been doubted. For example, the Scottish philosopher David Hume argued that there is no self as a mental entity. All there is is a complex set or ‘bundle’ of perceptions of interrelated events that reflect the world in its entirety. There is no additional self in the world; instead there is nothing but the events we perceive. Everything else, such as the assumption of a self as mental entity, is nothing but an illusion. The self as mental entity and thus as mental substance does not exist and is therefore not real.

The rejection of the self as mental substance and its outing as mere illusion is also currently popular. One major proponent of such view these days is the German philosopher Thomas Metzinger [1]. In a nutshell, he argues that through our experience, we develop models of the self, so-called ‘self-models’. These self-models are nothing but information processes in our brain. However, since we do not have direct access to these neuronal processes (e.g., all those processes and activities of the cells, neurons, in the brain) in our brain as neuronal processes, we tend to assume an entity that must underlie our own self-model. This entity is then characterized as self.

Following Metzinger, the assumption of the self as a mental entity results from a false positive and thus erroneous inference from our experience. We cannot experience the neuronal processes in our brain as such. Nobody ever experienced on-line his own brain as such and its neuronal processes. The outcome of our brain’s neuronal processes, the self, cannot then no longer be traced back to its original basis, the brain, in our experience. Where though does the self come from? We assume that it must be traced back to a special instance different from the brain. This leads us to assume a mind and the self as mental entity rather than as physical entity coming from the brain itself. Metzinger now argues that any such self as mental entity does simply not exist. Therefore, Metzinger [1] concludes, selves do not exist and can therefore be eliminated. Hence, the title of his book ‘Being no one’.

### Concept of self: from the metaphysical to the empirical self

What then is the self if not a mental entity? Current authors, such as Metzinger [1] and Churchland [2], argue that the self as mental substance or entity does not exist. How though do we come up with the idea of a self or the self-model as Metzinger says? The model of our own self is based on summarizing, integrating, and coordinating all the information from our own body and own brain. Take all that information together, coordinate and integrate it, and then you have a self-model of your own brain and body and their respective processes.

In more technical terms, our own brain and body are represented as such in the neuronal activity of the brain. And such representation is the model of your self. The self-model is therefore nothing but an inner model as the integrated and summarised version of your own brain and body’s information processing. The self is here thus a mere model of the own body’s and brain’s processes. The self, e.g., the self-model, consists then in nothing but a special form of representation.

**Figure 1:**
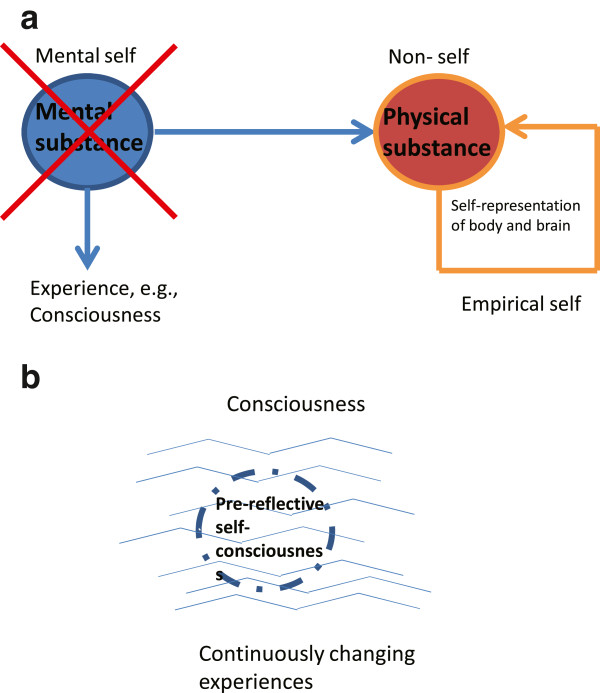
**Concepts of self. a** Mental self and its replacement by an empirical self. **b** Phenomenal self as pre-reflective self-consciousness.

The original mental self, the self as mental substance or entity, is here replaced by mere self-representation with a self-model. This implies a shift from a metaphysical discussion of the existence and reality of self to the processes that underlie the representation of body and brain as an inner model, e.g., as a self-model. Since such representation is based on the coordination and integration of the various ongoing processes in brain and body, it is associated with specific higher-order cognitive functions such as working memory, attention, executive function, and memory amongst others (Figure 1a).

What does this imply for the characterization of the self (presupposing a broader concept of self beyond the self as mental substance)? The self is no longer characterized as a mental substance but as a cognitive function. Methodologically, this implies that the self is no longer investigated in metaphysical terms with regard to its existence and reality. Instead, we need to search for the cognitive processes underlying the special representation implying empirical rather than metaphysical investigation. The question for the self is consequently no longer an issue of philosophy but rather one of cognitive psychology and ultimately of cognitive neuroscience. In short, the self is no longer a metaphysical matter but subject of empirical investigation.

### Concept of self: phenomenal self

Descartes ‘located’ the self outside the experience itself. His view of the self as mental substance is prior to and more basic than the experience itself. Only by ‘locating’ the self prior to and thus outside the experience, can the self make experience possible, e.g., consciousness. Experience and consciousness are thus presupposed by something lying outside itself. This outside is a mental substance as proposed by Descartes.

However, in phenomenological philosophy, such ‘outside’ location of the self is denied. Phenomenological philosophy is interested in investigating the structure and organisation of our experience and thus of consciousness. It focuses on how our experience is structured and organized and reveals phenomenal features as we experience them from the first-person perspective.

How does the phenomenal approach determine the self? Currently, it is argued that the self is an integral part of that very experience itself [3]. How can the self be part of our experience? The self is not present in the experience as distinct and separate content as is the case with objects, events, or other persons. Instead, it is always already present and manifest in the phenomenal features of our experience such as intentionality (e.g. the directedness of our consciousness towards specific contents), qualia (e.g., the qualitative character of our experience what it is like), etc. which without the self would remain impossible. Consequently, phenomenological philosophers such as Zahavi [4] (2005) describe as ‘pre-reflective self-consciousness’.

The concept of pre-reflective self-consciousness contains two main terms, ‘pre-reflective’ and ‘self-consciousness’. Pre-reflective means here that the experience of the self does not stem from any reflection or cognitive operation. Instead it is already always there as part of our experience such that we cannot avoid it. The self is thus pre-reflective. It is simultaneously an inherent part of our experience and thus of our consciousness. The self is consequently no longer outside of our consciousness but an integral part of it hence the second term, self-consciousness. Such an approach suggests an intimate and even stronger intrinsic linkage between self and consciousness (Figure 1b).

Characterizing the self in terms of self-consciousness implies a significant shift. The self is no longer metaphysical as in Descartes. Nor empirical as in Hume and advocates such as Metzinger and Churchland. Instead, the self is part of the experience and of consciousness itself and can therefore be characterized as the ‘phenomenal self’. Such a phenomenal self is thus open to systematic investigation of the phenomenal features of our experience which would complement the metaphysical, empirical, and logical approaches to the self.

### Concept of self: minimal self

How can we describe the pre-reflective self-consciousness in more detail? It is always already there in every experience so that we cannot avoid or separate it from the experience. The self is always present in our consciousness and thus in our subjective experience. Even if we do not focus on the self as such, we cannot avoid its presence. Hence, the pre-reflective self-conscious describes an implicit or tacit experience of our self in our consciousness.

Since the self as pre-reflectively experienced is the basis of all phenomenal features of our experience, it must be considered as basic and fundamental for any subsequent cognitive activity. Such basic and fundamental self occurs in our experience before any reflection. When for instance reading the lines of this book, you do experience the contents and in addition to that you also experience your self as reading these lines. Hence your immediate experience, e.g., consciousness, does come with both the content and your own self. Since the experience of such self occurs prior to any reflection and recruitment of higher-order cognitive functions, such self is sort of a minimal version of the self. Current phenomenological philosophers such as Gallagher [5] or Zahavi [4] speak therefore of a ‘minimal self’ when referring to the self as implicitly, tacitly, and immediately experienced in consciousness.

How can we describe the concept of the ‘minimal self’? The minimal self describes a basic form of self that is part of any experience. As such, it is not extended across time as it is case in the experience of a continuity of the self across time resulting in what is described as personal identity. Instead the minimal self describes a basic sense of self at any particular given moment in time. While it does not yet provide a linkage between different moments in time and thus a continuity across time.

How can such continuity across time be constituted? Cognitive functions such as memories and autobiographical memories in particular may be central here. The self may then become more complex and one may speak of a cognitive, extended or autobiographical self, as, for instance, the Portuguese-American neuroscientist, Damasio does (see [6,7]).

Another important feature of the minimal self is that although we experience it, we may not be aware of it as such nor able to reflect upon it in order to gain knowledge of it. We are, to put in technical terms, only pre-reflectively aware of the minimal self but not yet consciously, reflective aware of it as such. How can we become reflectively aware of the minimal self? That is possible when all different time moments are put together and, as philosophers say, represented as such. For such representation cognitive functions are needed which make possible the putting and linking together the different time points. By that the own minimal self is represented or reflected upon as minimal self – the corresponding functions can thus be termed self-representational functions as termed by Metzinger and Churchland.

Finally, the minimal self may also occur prior to and precede verbalization and thus linguistic expression. Rather than being tied to specific linguistic concepts as is the case with more cognitive concepts of the self, the minimal self must be considered pre-linguistic. It is an experience, a sense of self, that can barely be put into concepts. We can experience it as self but are not really able to describe these experiences in terms of concepts and thus in a linguistic way. Such minimal self is thus pre-linguistic and pre-conceptual. It may therefore occur predominantly in the unconscious mode rather than becoming conscious as such. The minimal self may thus be the subjective component of what Freud described as ego, the objective structure of our psyche. Future research will be needed to show the exact organisation and structure of the minimal self in order to reveal its psychodynamic relevance (see for instance [8,9]).

### Concept of self: social self

How does the self interact with other selves? So far we described the self by itself in an isolated and purely intra-individual way. However, in daily live, the self is not isolated from others but always already related to other selves and thus inter- rather than intra-individual. This raises the question for what is described as the ‘problem of other minds’ in philosophy or, more generally, the question for intersubjectivity.

How do we come to and make the assumption of attributing mental states and thus self and mind to other people? Philosophy has long relied on what is called the ‘inference by analogy’. What is the ‘inference by analogy’? The ‘inference of analogy’ goes like this. We observe another person A to show the behavior of type X. And we know that in our own case the same behavior, e.g. type X, goes along with the mental state type M. Since our own behavior and the ones of the person A are similar, i.e., behavior of type of X, we assume the other person A to show the same mental state type M we experience.

We thus infer from the analogy of behavior between us and the other person and our own associated mental states to the mental state of the latter. Hence, by indirect inference and analogy via our own case, we claim to obtain knowledge of the other person’s mental state. How can we make such inference? Very simple. We may make it on the basis of our own mental states and their associated behavior. And what we do may also hold for the other person who in the same attributes mental states to us by inferring them from the comparison between our behavior they observe and their own mental states.

Why do we make such inference? Because it seems to be the most easiest and best way for us to explain the others’ behavior. Only by assuming and inferring that you show mental states, I can explain your behavior. In other terms, your behavior of for instance taking the left street rather than the right one must originate in some kind of mental states that provides you with knowledge about the direction I, who chose the right street, do apparently not possess. The assumption of mental states thus seems to be the best explanation for your behavior. The ‘inference by analogy’ may thus be considered an inference to the best possible explanation.

The inference by analogy describes intersubjectivity in a very cognitive and ultimately linguistic way when attributing mental states and a self to other persons. There may though be a deeper level of intersubjectivity. We also feel the other persons’ mental states as for instance when sharing the emotional pain the spouse experiences when her father died. Such sharing of feeling is described as empathy and sheds the light on a deeper pre-cognitive and pre-verbal dimension of intersubjectivity as it has been emphasized in especially phenomenological philosophy (see for instance Zahavi [4] (2005)).

Both, empathy and the attribution of mental states to another person are however slightly puzzling: despite that the fact that we do not experience the other’s mental states and consciousness, we nevertheless either share them (as in empathy) or infer them (as in the inference by analogy). We have no direct access to other persons’ experience of a self and its mental states in first-person perspective and nevertheless share their mental states and assume that they have a self. How is that possible?

This is the moment where we need to introduce yet another perspective. There is the first-person perspective that is tied to the self itself and its mental states, the experience or consciousness of objects, events, or persons in the environment. And there is the third-person perspective that allows us to observe the objects, events, or persons in the environment from the outside rather than from the inside as in the experience in first-person perspective. The picture however is not complete yet. The interaction between the different selves as well as the second-person perspective as sandwiched between first- and third-person perspective are missing here.

What is the Second-Person Perspective? The Second-Person Perspective has initially been associated in philosophy with the introspection of the own mental states. Rather than actually experiencing the own mental states in first-person perspective, the second-person perspective makes possible to reflect and introspect about the own mental states. That is for instance the case when we ask ourselves whether it is really true that I heard the voice from another person speaking out there in the environment (see also Schilbach et al. [10] 2013).

The second-person perspective thus allows to put the contents as experienced in first-person perspective into a wider context, the context of the own self as it is related to the environment. In other terms, the second-person perspective makes possible to situate and integrate the purely intra-individual self with its first-person perspective into a social context thereby transforming it into an inter-individual self. One can thus say that the concept of self is here determined in a social way so that one can speak of a ‘social self’ ( [10,11]).

How can we define the concept of the social self? The concept of the social self describes the linkage and integration of the self into the social context of other selves. This shifts the focus from the experience or consciousness in the first-person perspective of a sole self to the various kinds of interactions between different selves as associated with the second-person perspective. As we already indicated there may be different kinds of social interactions including pre-cognitive and more cognitive ones.

## Empirical account of self

We so far described the self in purely conceptual terms as it is discussed in philosophy. This however leaves open the empirical characterization of the self, more specifically the exact mechanisms that give rise to what we described as self in the different conceptual facets. Obviously the brain is central making possible the constitution of a self. How though can we investigate the self empirically in neuroscience? This is the question for the kind of methodological strategies that we can apply to experimentally investigate the self and its relation to the brain’s neuronal mechanisms.

### Empirical account of self: methodological approaches to the experimental investigation of the self

How can we investigate the self? In order to experimentally address the self, we need some quantifiable and objective measures that can be observed from a third-person perspective as distinguished from subjective experience from the first-person perspective. How can we obtain such measures? Psychologists focusing on memory observed that items related to the own person were better remembered, e.g., recalled, than those unrelated to the person (see [12]). For instance, living in Ottawa, I recall much better the recent thunderstorm that wiped away several houses locally than you do as the reader, perhaps living in Germany, who just heard about it in the news.

There is thus superiority in recall of those items and stimuli that are related to one’s own self. This is described as self-reference effect (SRE). The SRE has been well validated in several psychological studies [12]. Most interestingly, it has been shown to operate in different domains. Not only in respect to memory but also in relation to emotions, sensorimotor functions, faces, words, etc. In all these different domains (see below for details), stimuli related to one’s own self, e.g., self-specific stimuli, show much better recall than those that are unrelated to one’s own self, e.g., non-self-specific stimuli.

How is the SRE possible? Numerous investigations (see, e.g., [13,14] for summaries) show that the SRE is mediated by different psychological functions. These range from personal, e.g., autobiographical memories over memories of facts, e.g., semantic memories, to those cognitive capacities that allow for self-reflection and self-representation as introduced above, e.g., representing the processes in one’s own brain and body. Hence, the SRE is by itself not a unitary function but rather a complex multifaceted psychological composite of functions and processes.

How can we link the SRE to the brain? Before the introduction of functional imaging techniques like fMRI at the beginning of the 1990s, most studies conducted focused on the effect of dysfunction or lesions in specific brain regions by for instance brain tumors or stroke. These revealed that, for instance, lesions in medial temporal regions that are central in memory recall, such as the hippocampus, change and ultimately abolish the SRE effect.

With the introduction of brain imaging techniques such as fMRI, we could then transfer the experimental paradigms of comparing self- and non-self-specific stimuli to the scanner and investigate the underlying brain regions. The basic premise here is that if self-specific stimuli are better recalled than non-self-specific ones, they must be processed in the brain in a different way such as, for instance, by higher degrees of neural activity and/or different regions.

This led to the investigation of numerous experimental designs of SRE-like paradigms in the fMRI scanner. For example, subjects were presented trait adjectives that were either related to themselves (such as for me, my hometown, Ottawa) as opposed to (Sydney, an unrelated city for me). Or the participant’s own face was presented and compared with faces of other people. Also autobiographical events from the subject’s past were compared with those from other people. One’s own movements and actions could also be compared with those of other people implying what is called ownership (e.g., my movements) and agency (“I my self caused that action’).

As can be seen, the stimuli belonged to different domains such as memory, faces, emotions, verbal, spatial, motor, or social. Most of the stimuli were presented either visually or auditorily. Also the presentation of these stimuli was most often accompanied by an on-line judgment about whether the stimuli are related, e.g., personally meaningful, or not to the respective subject.

### Empirical account of self: spatial patterns of neural activity during self-reference

What results did the various imaging studies yield in fMRI? Two different kinds of regions showed. First, one could see that the regions specific for the respective domain like emotions or faces were recruited. For instance, there is a region in the back of the brain that processes specifically faces (as distinguished from say houses), this is called the fusiform face area. This region is obviously active during the presentation of faces no matter whether it is one’s own face or another person’s face. Importantly, clear differences between self- and non-self-specific stimuli could not be observed in these domain-specific regions in most of the studies (see [12]).

What about other regions that are not specific for a particular domain like emotions or faces, e.g., domain-independent regions are involved in the neural processing of the self? ? Meta-analyses of the various studies demonstrated the involvement of a particular set of regions in the middle of the brain. These regions include the perigenual anterior cingulate cortex (PACC), the ventro- and dorsomedial prefrontal cortex (VMPFC, DMPFC), the supragenual anterior cingulate cortex (SACC), the posterior cingulate cortex (PCC) and the precuneus. Since they are all located in the midline of the brain, they have been coined ‘cortical midline structures’ (CMS).

**Figure 2:**
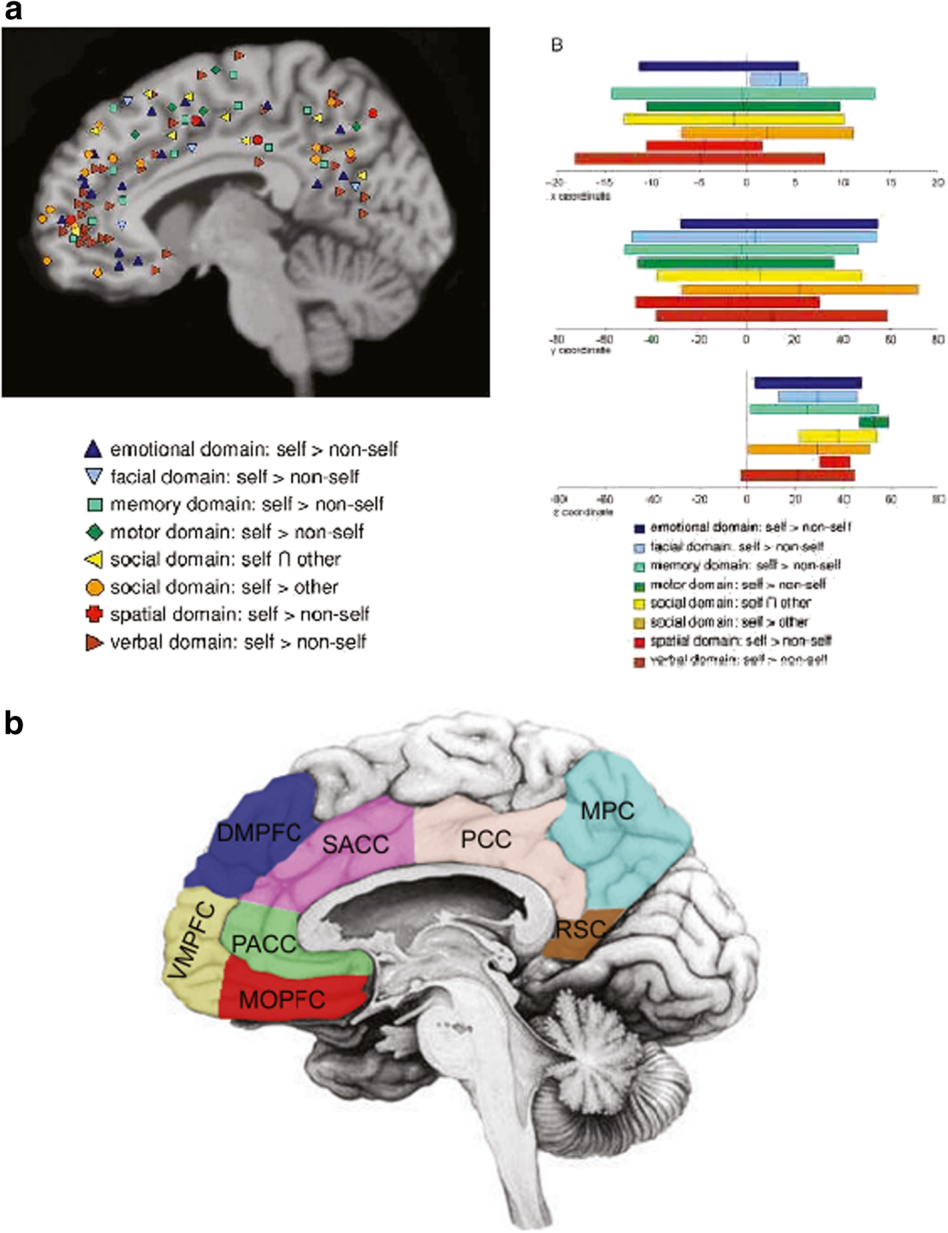
**Cortical midline structures and the self. a** Distinction between self and non-self: cortical midline structures and domain indepedence. **b** Cortical midline structures-anatomical definition.

The self-specific stimuli, e.g., those that were personally relevant for the subjects, induced higher neural activity in these regions than non-self-specific ones, e.g., those that remained irrelevant und unrelated to the person. This was observed in the various domains for faces, trait adjectives, movements/actions, memories, and social communication. The CMS thus seem to show a special significance to the self, e.g., self-reference (Figure 2).

There is some differentiation within the CMS though. The self-specific stimuli may be presented in different ways to the subject in the scanner. If subjects have to make judgments requiring cognitive involvement, the dorsal and posterior regions like the SACC, DMPFC and PCC are recruited to a stronger degree. If, in contrast, stimuli are merely perceived without any judgment and thus cognitive component, the ventral and anterior regions like the VMPFC and PACC were strongly involved.

This led to the assumption that the different regions mediate different aspects of self-reference. The ventral and anterior regions such as the PACC and VMPFC may be more involved in the representation of the stimulus’ degree of self-reference. However, dorsal regions such as the SACC and the DMPFC may be related to monitoring and reflection of the stimulus and its self-reference when we become aware of the stimulus as self-specific. Finally, the posterior regions like the PCC may be implicated in integrating the stimulus and its degree of self-reference into the autobiographical memory of the respective person. These regions have been shown to be implicated in the recall and retrieval of especially personally relevant and thus autobiographical information from the past of that person.

In sum, specific regions in the midline of the brain, the cortical midline structures, seem to be involved in the neural processing of self-reference, e.g., attributing personal relevance or self-relevance to stimuli.

### Empirical account of self: temporal patterns of neural activity during self-reference

In addition to the spatial patterns of self-reference, its temporal patterns have also been investigated using electroencephalogram (EEG) for the measurement of electrical activity in the brain. Again self- and non-self-specific stimuli have been compared with each other while the subjects undergo EEG measurement. This revealed early changes during self-specific stimuli at around 100-150 ms after stimulus onset, e.g., the beginning of the stimulus.

Self-specific stimuli induce earlier temporal changes at 130-200 ms after the beginning of the stimulus than non-self-specific changes in the brain’s electrical activity. Moreover, self-specific stimuli induce later changes at around 300-500 ms. Hence, the temporal pattern between self- and non-self-specific stimuli shows early and late differences. Neural activity is variable and oscillates. These oscillations occur in different frequency ranges. One such frequency range of the Gamma frequency occurring in a range of 30-40 Hz. Some studies now observed that self-specific stimuli induce stronger oscillations in the Gamma frequency, e.g., stronger power, than non-self-specific stimuli which occurred especially in the midline regions. However, functions other than self-specificity like sensorimotor functions or cognitive functions like attention or working memory also go along with higher increases in the Gamma frequency range which therefore may be considered unspecific to the self.

### Empirical account of self: social patterns of neural activity during self-reference

How can we investigate the earlier described social nature of the self? Various studies have been conducted to investigate different kinds of interaction between different selves. Pfeiffer et al. [11] (2013) and Schilbach et al. (2013) [10] distinguish two different methodological approaches. One can investigate social cognition, the cognition of other people’s mind, from the outside and thus from an observer’s point of view. Social cognition is here investigated in an “offline” mode. More recently such “offline” methodological strategy has been complemented by an “online” mode. Here the social interaction is no longer investigated from the “outside” but rather from the “inside” by taking the perspective or point of view of the interacting selves themselves (rather than the observer’s point of view).

Besides conducting several studies, the same group now recently investigated the neural overlap between emotional processing, resting state activity, and social-cognitive processing (Schilbach et al. [15] 2012). They conducted a meta-analysis including imaging studies from all three kinds of investigations, resting state, emotional, and social-cognitive. In a first step they analysed the regions implicated in each of the three tasks. This yielded significant recruitment of neural activity in especially the midline regions like the ventro and dorsomedial prefrontal cortex and the posterior cingulate cortex (bordering to the precuneus). In addition, neural activity in the temporo-parietal junction and the middle temporal gyrus was observed.

In a second step they overlaid the three tasks, emotional, social-cognitive, and resting state, in order to detect commonly underlying areas. This indeed revealed the midline regions, the dorsomedial prefrontal cortex and the posterior cingulate cortex, to be commonly shared among emotional and social-cognitive tasks and the resting state activity. Based on this neural overlap the authors conclude that there may be an intrinsically social dimension in our neural activity which may be essential for any subsequent consciousness of both our own self and other selves. If this holds, it will have rather radical consequences not only for the concept of the self but for consciousness in general as we will indicate at the very end of this paper.

## Neurophilosophical reflection

How can we now link the empirical data about self-reference from neuroscience to the conceptual determinations of the self in philosophy? One way is to directly infer the concept of self from the empirical data as it is for instance suggested by the earlier described proponents of an empirical self. That however is to neglect that empirical and conceptual domains do not need to correspond one-to-one. Instead, the conceptual domain and its definition of the self may go beyond the data in the empirical domain (or vice versa). Due to such possible difference between empirical and conceptual domains we need to investigate the degree of correspondence or matching between empirical data and conceptual definitions of the self. In other words, we must investigate the empirical plausibility of the conceptual definitions in order to yield a truly neurophilosophical concept of self.

### Neurophilosophical reflection: Psychological and experimental specificity

How can we directly compare empirical data and conceptual definitions? Before making their direct comparison, we need to be clear about the empirical data themselves. What exactly can they tell us about the self? This touches upon the question how specific the obtained data are for the self as distinguished from other psychological and mental features. In other words, we have to check for the specificity of the data which may take on different forms.

Most of the FMRI and EEG studies above compared self- versus non-self-specific stimuli, such as a grand piano for a professional pianist compared to a saw for a carpenter. In addition to the mere perception, subjects were required to make a judgment after each stimulus, to judge whether it was self- or non-self-specific. This raises the question about what the study is measuring - the perception or the judgment of the stimulus? Is it thus capturing the effect of the stimulus itself or the task related to that stimulus?

Most likely the results reflect a mixture between stimulus- and task-related effects. This therefore sheds some doubt on whether the midline regions show psychological specificity for the self. The judgment about self-specificity requires various cognitive functions such as attention, working memory, and autobiographical memory retrieval. Some authors, such as the French neuroscientist D. Legrand [16], therefore argued that the midline regions may be more related to what she describes as ‘general evaluation function’, rather than being specific to the self and self-specific stimuli.

What about when research investigates self in relation to more basic functions such as movements and actions? Even when subjects perform some motor tasks, we face the same confusion of different functions: The self components such as ownership, e.g., is the movement my own movement, as well as agency, e.g., whether I am the agent of that very movement, may be confounded by the neural mechanisms underlying the execution of the movement/action by the person.

Such psychological unspecificity highlights the need in neuroscience to specify the experimental design and measures. We need measures that are specific to the self as distinguished from the various associated sensorimotor, affective, and cognitive functions. And we need experimental designs to segregate stimulus- and task-related effects, for example, by spacing perception and judgment temporally apart from each other.

### Neurophilosophical reflection: Self-specificity and other functions

Finally, one needs to discuss the relationship between self and other functions. Recent imaging studies demonstrated strong neural overlap between self and reward, self and emotions, and self and decision making. For example, when receiving a reward in relation to a specific stimuli, such as money, regions of the reward system like the ventral striatum (VS) and the ventromedial prefrontal cortex (VMPFC) become active [17]. These same regions are also very active when the same stimulus is conceived as self-specific rather than non-self-specific by the respective subject. The same effects can be observed in emotions where emotional and self-specific stimuli have been shown to overlap in especially the anterior midline regions, such as the perigenual anterior cingulate cortex and the VMPFC.

Finally, the same can be observed in decision making: If external cues are provided when making a decision (such as a higher or lower price of the same kind of apples), lateral cortical regions become active. If, in contrast, no such external cues are provided, we need to come up with some internal criterion to guide and make our decision about which to purchase [18]. Such internal criterion can only stem from our self. Studies comparing both kinds of decision making showed predominant involvement of the midline regions in internally-guided when compared to externally-guided decision making [18].

Together such neural overlap between self and other functions such as reward, emotions, and decision making raises questions about the relationship between them. Different models could be imagined. Self- and self-specificity could be an independent function just like attention, working memory, emotion, sensorimotor, etc. However, in that case, one would expect specific regions in the brain and specific psychological functions to subserve specifically and exclusively self-specificity. This though at this point in time, cannot be supported empirically.

Finally, one could also suggest that self and self-specificity are basic functions that underlie and provide the basis for all other functions, e.g., sensorimotor, affective, cognitive, social. In this sense, self and self-specificity would occur prior to the recruitment of the other functions. Self-specificity would then always be there making its involvement and manifestation in the various functions unavoidable. Rather than searching for self-specificity in relation to specific cognitive functions such as language, one would then need to look for more basic functions that must occur prior to the other ones.

Self-specificity in that sense may then also be linked to psychodynamically relevant mechanisms like defense mechanisms that then may describe the structuring and organising of the content in relation to the respective self. Hence, self-specificity may then be put into psychodynamic context in order to better understand its structure and organisation and its mechanisms of operation as presumably manifest in defense mechanisms (see [8]). Thereby one may assume neuronally the interplay between subcortical and cortical midline structures to be central which though remains to be researched in detail (see [8,9]).

### Neurophilosophical reflection: Phenomenal specificity of the self

To recap, the minimal self describes a basic sense of self that occurs immediately and is always already part of our experience of the world. The question now is how the concept of the minimal self is related to the neuroscientific results discussed above. For that we briefly have to shed a light on the experience of the minimal self as manifest in pre-reflective self-consciousness.

Consciousness can be characterized by various phenomenal features like qualia and first-person perspective. In short, qualia describes the point of view and its what it is like of our experience. All your experience presuppose a specific point of view, your individual one, that is different from the one of other persons. This individually specific point of view is supposed to give your experience a specific quality, qualia. The first-person perspective refers to the fact that we can experience the world only from a first-person perspective while any experience remains impossible from a third-person perspective where we can only observe but not experience.

If the self, e.g., the minimal self, is part of any experience (rather than being outside of it), the self should be manifest in these phenomenal features too. One may therefore consecutively speak of self-qualia or first-person giveness of the self as phenomenological authors [4,5] do. What experiential and thus phenomenal features does the self add?

Phenomenological philosophers assume that the special contribution of the self consists in what they describe as ‘belongingness’ or ‘mineness’ [4,5]: The contents of our experience are experienced as belonging to a particular self, they are experienced as mine. For instance, I experience the laptop on which I write here in front of me as my laptop going along with an experience of mineness or belongingness. However, such experience is not possible for the person sitting besides me who though looking at the same laptop does not experience any mineness or belongingness. Instead, he may experience mineness or belongingness of the CD lying besides the laptop because he is a composer and it is a CD of his work.

Such mineness or belongingness are particularly important when relating to specific contents which make up our unconscious. Even unconscious contents may induce mineness and belongingness the degree of which may then signal how much they will occupy the unconscious and be relevant in future thoughts and behaviour. In other terms the concepts of mineness and belongingness provide a real bridge to mechanisms discusses in psychoanalysis and, more specifically, defense mechanisms. Defense mechanisms may be particularly strongly recruited in case of unconscious contents with a high degree of mineness and belongingness while they may not be set in motion if the relevance is low. In short, the concepts of mineness and belongingness may be psychodynamically highly relevant.

## Neurophilosophical conclusion

What do these considerations tell us about the self? We here could not conduct a full-blown neurophilosophical investigation of the self which is beyond the scope of this paper. What however we were able to do is to show what information the empirical data contain about the self when discussing the issue of the various forms of specificity. What kind of self shall now we opt for? Which of the various concepts of self as discussed in philosophy is empirically plausible? We will not be able to reach a neurophilosophical conclusion but can only indicate a couple of points that may be important for future neurophilosophical investigation.

### Neurophilosophical conclusion: Phenomenal specificity and phenomenal limits

In order to account for phenomenal specificity, neuroscience needs to show the neuronal mechanisms underlying the experience of mineness and belongingness and also to distinguish those neuronal mechanisms underlying the other phenomenal features of experience, intentionality, unity, first-person perspective, qualia, and spatiotemporal continuity. One would therefore require distinct experimental measures and designs for each of these phenomenal features. Only then would we be able to achieve phenomenal specificity and to clearly distinguish the phenomenal or minimal self from phenomenal consciousness. In short, we need to experimentally distinguish between self- and non-self-specific phenomenal measures.

However, the phenomenological philosopher may want to raise the following question: Is such phenomenal specificity with the experimental distinction between self and non-self-specific phenomenal measures really possible at all? The minimal self is considered part of the experience and thus of consciousness in general. Any consciousness of the world goes along with an experience of the self in a pre-reflective way. And the converse holds too. Any experience of the self is part of an experience of the world. Both experience of self and experience of world are thus intrinsically linked.

What does such an intrinsic link between experience of self and experience of the world imply for the phenomenal specificity of the self? It means that we will remain unable to properly and clearly segregate experimental measures for the minimal self from those of our experience in general, e.g., experience of the world. More specifically, this means that we will be unable to account experimentally for mineness and belongingness distinct and separate from other spatiotemporal features such as spatiotemporal continuity, unity, first-person perspective, and qualia.

Why? Because these phenomenal features are always already ‘infected’ by the self, e.g., mineness and belongingness, in the same way as they are encoded and ingrained into the self.. Hence, the requirement of maximal experimental and phenomenal specificity may here have reached its phenomenal limits. If so, we may be forced to acknowledge that there may be principal limitations in what we can and cannot investigate experimentally when it comes to the minimal self.

### Neurophilosophical conclusion: Minimal self and body

How about self and body? We can experience our own body as our own body. This leads us to the characteristic feature of the body, namely, that it can be experienced by itself in consciousness. The body is not only an objective body that can be observed from a third-person perspective. This is the body the neuroscientist and the doctor investigate. It can also be experienced from a first-person perspective. This is the body we experience in consciousness which is therefore characterized as a ‘lived body’.

The ‘lived body’ is the body which we experience as our body, as my body as distinguished from others’ bodies. Hence, we experience the lived body in terms of mineness and belongingness. Thus, the experience of the body, the lived body, may be regarded as the first and most fundamental manifestation of the phenomenal or minimal self. Our self in its most basic and minimal form is thus essentially a bodily self.

Such mineness and belongingness is also reflected in what we described earlier as ownership and agency. Ownership describes that I experience my body as my body rather than some other body. Neuroscientifically, the ownership of the body has been associated with neuronal activity in specific regions of the brain such as the sensory cortex and the parietal cortex with the parietal cortex mediating the spatial position of the body in the world.

Agency is the experience that I rather than some other person originated and caused the subsequent action and movement. I, e.g., my self, am the agent of the lines I am currently writing here on my laptop. The action and the movements are thus mine since they were caused by me as agent. Neurally, regions such as the premotor cortex and the motor cortex have been associated with agency; these are regions that are implicated in generating movement and action in general.

### Neurophilosophical conclusion: Self as brain-based neurosocial structure and organization

What does this imply for the self? Our self may be considered as intrinsically to the body thus being embodied. Furthermore, since it is based on self-reference as the attribution of personal relevance to environment (and bodily stimuli), our self may also be intrinsically linked to the environment thus being embedded and social. Our self can consequently not be regarded an entity located somewhere in the brain and isolated from both body and environment. Instead our self seems to be intrinsically social as suggested by the advocates of the concept of a social self (see earlier).

What does such intrinsically bodily and social nature imply for the conceptual characterization of the self? Our self may be described as structure and organisation rather than as entity be it mental or physical. Such structure and organisation needs to develop through childhood and adolescence with persistent changes even throughout adulthood. Despite all the changes there may be persistence and continuity across time which then accounts for what can be described as identity. Identity may describe the persistence and continuity of self over time which, in an exploratory study, has recently been associated with the midline structures and their high intrinsic activity (see [19]).

We can also see that such concept of self as structure and organisation is embodied, e.g., intrinsically linked to the body, and embedded, e.g., intrinsically linked to the environment. Hence, the virtual structure of the self spans across brain, body, and environment with the brain’s midline structure activity being a neural predisposition for its constitution, while at the same time being dependent upon the respective environmental context. Freud’s characterization of the ego as structure and organisation thus surfaces here in a more specific embodied and embedded gestalt as intrinsically relational or biopychosocial. Future investigation may now link the different features Freud attributed to the ego to the self as understood here and the above described mechanisms (see [8,9]).

What however do we mean exactly by the concepts of structure and organization? The structure must be virtual in that it spans across the physical boundaries of brain, body, and environment. Does this mean that we have to revert to a mental structure and organization as distinct from the physical structure and organization of the brain? No! The results from neuroscience clearly link the self with neuronal processes related to both intraindividual experiences and interindividual interaction. There is thus a neuronal basis for the distinct aspects of the self within the context of brain, body, and environment. We therefore reject the mental characterization of the structure and organization that is supposed to define the self.

How can we define the concepts of structure and organization in a more positive way? One way is to characterize structure and organization as social as distinguished from both mental and physical. The social characterization would then be an intermediate or better commonly underlying basis between the purely physical and the purely mental. The self is then based on the brain but extends beyond it to body and environment. This means that conceptually, we need to characterized the concept of the self as brain-based rather than brain-reductive (as the proponents of the empirical self tend to do). Such brain-based nature of the self also excludes both mind-and consciousness-based approaches to the self as advocated by the earlier philosophical approaches in their concepts of a mental self and a phenomenal self.

If the social characterization of the structure and organization as related to the self is indeed as basic and fundamental, one would assume that our brain’s neural activity is intrinsically social [15] that is neurosocial by default: the brain cannot avoid including the social environmental context in the encoding of stimuli into its own neural activity which then is by default neurosocial rather than purely neuronal [20]. This is supported by the earlier described neural overlap between resting state activity and the neural activity changes during emotional and social-cognitive tasks.

Whether the brain encodes its neural activity indeed in an intrinsically neurosocial way remains however unclear at this point. What is clear though is that the exact characterization of the brain’s neural activity will be essential to develop a truly neurophilosophical (rather than philosophical or neurocientific) and thus brain-based (rather than brain-reductive) and neurosocial (rather than merely neuronal) concept of the self.
